# Unexpected rhythm regularity in a patient with atrial fibrillation and a changing frontal plane axis over time

**DOI:** 10.1007/s12471-016-0912-9

**Published:** 2016-10-26

**Authors:** A. Zweerink, E. Leusveld, C. P. Allaart, M. J. B. Kemme

**Affiliations:** Department of Cardiology, VU University Medical Center, Amsterdam, The Netherlands

A 73-year-old women receiving digoxin for permanent atrial fibrillation was admitted to the intensive care unit with respiratory failure due to a bacterial pneumonia, several weeks after coronary artery bypass grafting with aortic and mitral valve replacement. Since her ECG showed atrial fibrillation with rapid ventricular rates, an additional dose of digoxin was given for rate control. The same night she developed a regular tachycardia (Fig. 1) with a changing frontal plane axis over time (Fig. 2). Electrical cardioversion was unsuccessful. What is the aetiology of the tachycardia? And what causes the changing frontal plane axis over time? How should this patient be treated?

**Fig. 1:**
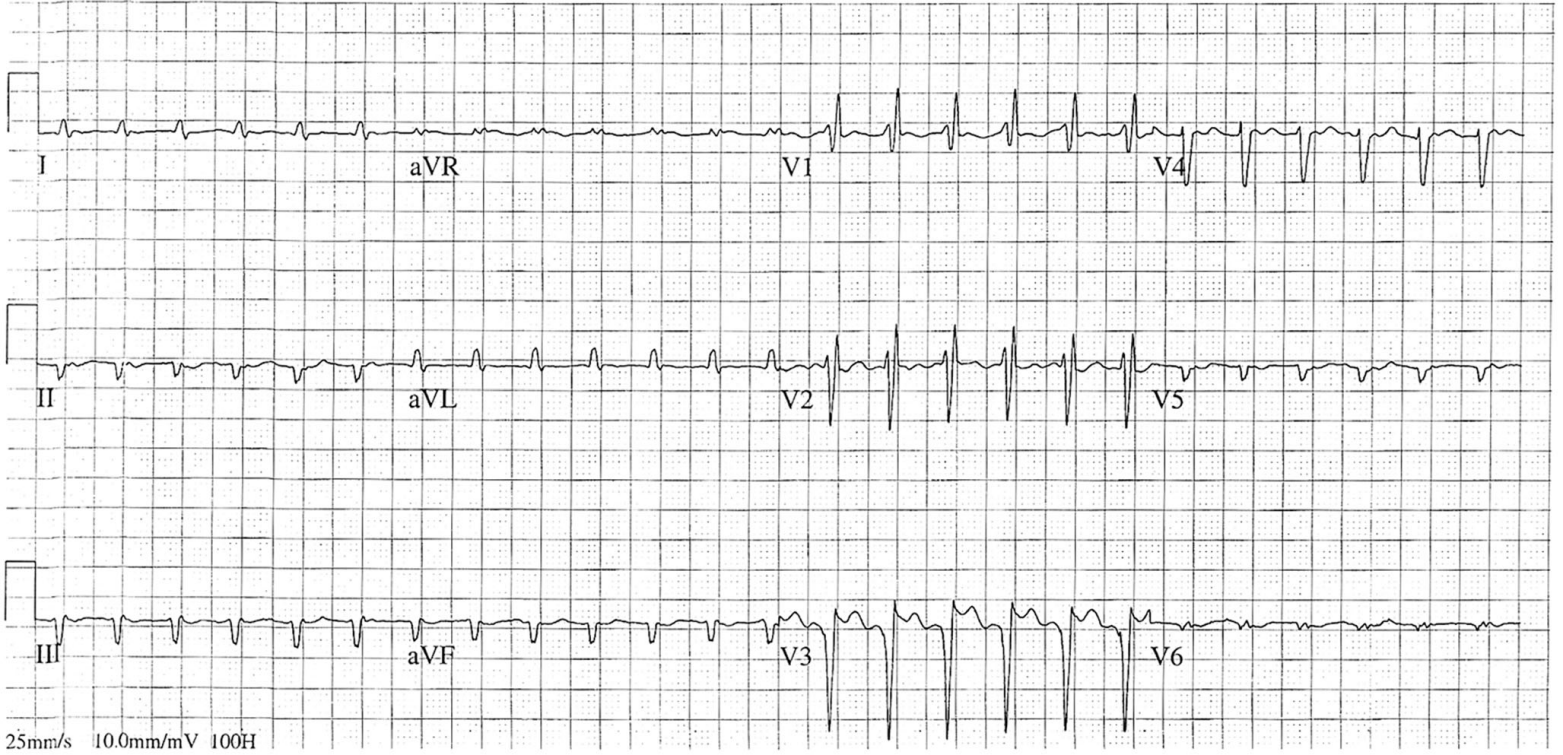
Unexpected rhythm regularity in a patient with atrial fibrillation

**Fig. 2:**
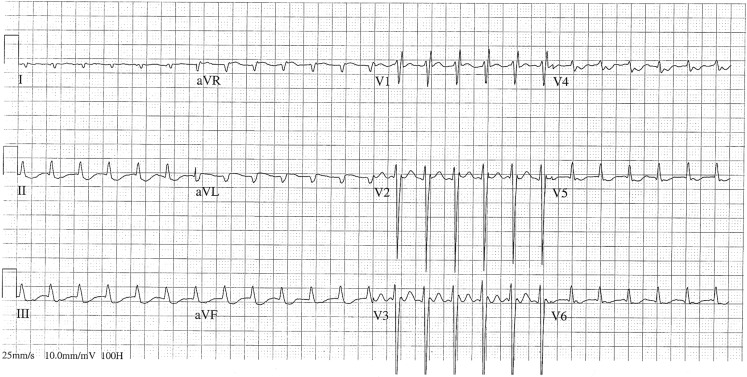
Changing frontal plane axis over time

## Answer

You will find the answer elsewhere in this issue.

